# Matrine-loaded self-adhesive swelling microneedle for inflammation regulation to improve eczema treatment

**DOI:** 10.1007/s42995-024-00235-z

**Published:** 2024-06-26

**Authors:** Jiale Shen, Jiarui Wang, Meng Wu, Yan Shi, Minhyeock Lee, Zhiguo Wang, Ming Kong

**Affiliations:** 1 College of Marine Life Sciences, Ocean University of China, Qingdao, 266003 China; 2 Department of Burn and Plastic Surgery, the Affiliated Hospital of Qingdao University, Qingdao University, Qingdao, 266021 China; 3 Department of Biotechnology, College of Life Sciences and Biotechnology, Korea University, Seoul, 02841 Republic of Korea

**Keywords:** Self-adhesive swelling microneedle, Eczema, Matrine, Inflammation regulation

## Abstract

Eczema is a common chronic dermatological disease. Conventional treatments exhibit limited efficacy due to fast drug release resulting in short-term relief. Development of a new treatment strategy that enables sustained drug release and long-term maintenance on the skin surface is necessary. A self-adhesive swelling microneedle patch (SDSMNs) was designed and constructed using a two-step casting method. The adhesive substrate was prepared by blending gelatin and dopamine via oxidation of NaIO_4_, so it could adhere onto the skin surface as well as withstand repeated bending movement without detachment. The swelling needles were fabricated using polyvinyl alcohol (PVA) and polyvinylpyrrolidone (PVP), which could swell by absorbing interstitial fluid and release the drug in a controlled manner. SDSMNs also showed desirable antibacterial activities toward *E. coli* and *S. aureus*. The adhesive microneedles loaded with matrine (MAT-SDSMNs), an anti-inflammatory Chinese medicine, dramatically relieved eczema symptoms through IL-17 mediated inflammation responses. The use of MAT-SDSMNs significantly decreased the infiltration of inflammation cells and level of inflammatory cytokines, reduced the skin thickness, and increased collagen deposition fraction compared with conventional ointment or subcutaneous injection. The results suggested that MAT-SDSMNs can improve eczema treatment by regulating the local inflammatory microenvironment, providing a simple, self-administered sustainable strategy for eczema treatment.

**Supplementary Information:**

The online version contains supplementary material available at 10.1007/s42995-024-00235-z.

## Introduction

Eczema (synonymous with atopic dermatitis) is a frequently occurring chronic inflammatory skin disease that is heterogeneous and highly variable. It is mainly characterized by dry skin, severe itching, inflammatory skin lesions, long duration, and difficult to cure (Andersen et al. 2016; Eichenfield et al. 2014; Weidinger and Novak 2016). Additionally, eczema sometimes presents as red, irritating, and scaly plaques, severely impacting the quality of life for those afflicted (Nutten 2015).

The pathogenesis of eczema is attributed to multiple factors, such as genetics, allergies, immune mediators, physical psychology, living environment, among others (Dourmishev and Mironova 2021; Flohr and Mann 2014; Mandlik and Mandlik 2021). An immune disorder caused by the imbalance of Th1/Th2 (helper T lymphocytes) is the main mechanism of eczema onset. Related immune cells and mediators serve as key regulatory factors to mediate the disease by regulating relevant immune responses. Activated Th1 cells secrete a large number of inflammatory factors, activating macrophages at inflammatory sites, enhancing the resistance of host antigens, and inducing delayed hypersensitivity (Wang and Landén 2015). The inflammatory factors promote the cascade reaction of inflammation, inducing release of tumor necrosis factor-α (TNF-α), interleukin-1 β (IL-1β), and interleukin-6 (IL-6), exacerbating skin lesions (Azizi et al. 2015; Nguyen et al. 2015; Noda et al. 2015). Additionally, Th17 immune regulatory factors have important effects on eczema. IL-17 activates the nuclear factor kappa-B (NF-κB) signal transduction pathway in target cells. It increases the expression of intercellular adhesion molecule intercellular cell adhesion molecule-1 (ICAM-1) and mitogen-activated protein kinase (MAPK) by stimulating target cells to produce inflammatory cytokine and chemokines, leading to the occurrence of inflammatory reactions and tissue damage (Ahmad et al. 2017; Hofmann et al. 2016; Puig 2017). Hence, regulation of the local inflammatory microenvironment is necessary to improve existing eczema treatments.

Reducing inflammation by inhibiting the release of inflammatory factors when treating eczema is a necessary measure to treat eczema. Drug therapy is a highly effective and widely used approach, including anti-infective, hormone, and anti-allergic drugs (Carello et al. 2017; Martinovich and Baradzina 2022). Matrine, a type of traditional Chinese medicine can reduce the secretion of various inflammatory factors by inhibiting the expression of inflammatory signal pathways to achieve the effect of anti-inflammation (Sun et al. 2022). At the same time, matrine can also exert antibacterial, antipruritic, and immune regulatory effects, as has been demonstrated in clinical practice to treat skin, liver, gynecological, respiratory, and other related diseases (Zhang et al. 2020). Due to the local presence of eczema, topical transdermal administration is an essential part of treatment. Conventional administration formulations, such as ointment or subcutaneous injection, generally lead to fast drug release but short-term relief, especially for chronic symptoms. Kim et al. (2022) developed a therapeutic hydrogel patch for atopic dermatitis treatment that could extend the drug release period along with providing a dressing for wound repairment. However, the hydrogel patch can detach from the skin surface, especially at joint sites. Microneedles are a promising alternative to treat eczema. They have been used to treat other skin diseases. Microneedles are characterized by their high transdermal drug delivery efficiency (Yang et al. 2019), controlled drug release (Shi et al. 2022), and modulation of topical tissue microenvironment (Zhou et al. 2020).

**Fig. 1:**
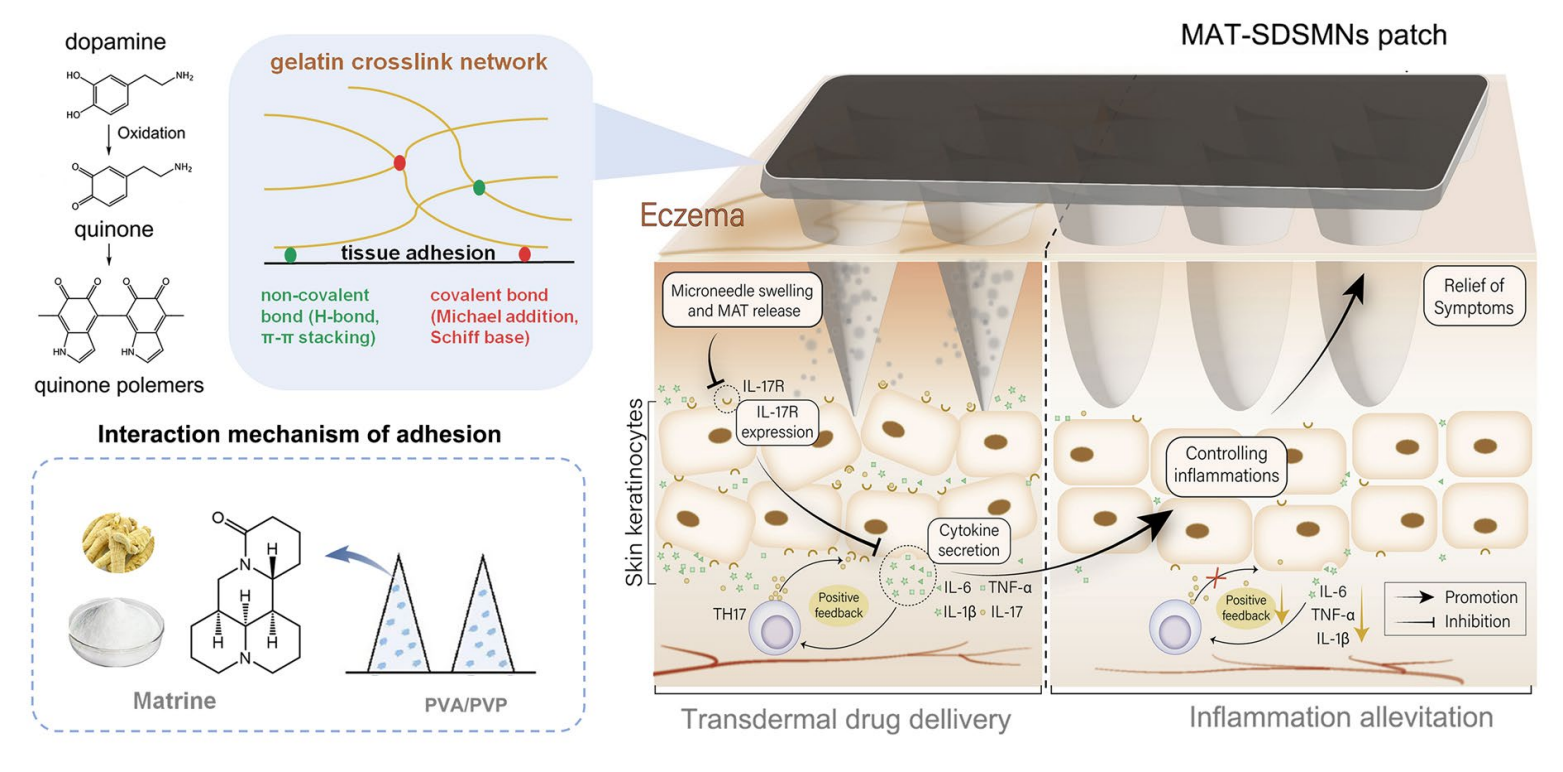
Schematic of the construction and application of matrine-loaded self-adhesive swelling microneedle patch (MAT-SDSMNs) used to treat eczema. The adhesive substrate of MAT-SDSMN was prepared using dopamine and gelatin oxidized by NaIO_4_. The catechol groups of dopamine are oxidized to be quinone groups that are reactive with thiol and amine groups via Michael-type addition or Schiff-base formation reactions, along with the hydrogen bond and π–π stacking. This results in the crosslink with gelatin and adhesion on tissue interface. The swelling needles are comprised of PVA and PVP, which could swell by absorbing interstitial fluid after insertion into skin. Matrine could alleviate local inflammation to improve eczema treatment (colour figure online)

In this study, a self-adhesive swelling microneedle (SDSMNs) was designed and developed. The microneedles were prepared using polyvinyl alcohol (PVA) and polyvinylpyrrolidone (PVP). The substrate was prepared from dopamine and gelatin via covalent and non-covalent cross-linking for eczema treatment (Fig. 1). After inserting into the skin, the needles swell by absorbing interstitial fluid and then release the loaded matrine in a controlled manner. The substrate would be hydrated to generate adhesion on tissue interfaces through physical and chemical bonding between oxidized dopamine and the reactive groups on tissue surface, allowing for long-term attachment of SDSMNs on skin. In addition, their potential antibacterial activity could also prevent infection during application. The therapeutic effects on eczema cell and animal models were studied from the perspective of inflammatory microenvironment regulation to improve eczema treatment.

## Materials and methods

### Material

Pigskin gelatin (Type A, G-A) and cattle skin gelatin (Type B, G-B) were purchased from Shanghai Maokang Biotechnology Co., Ltd. Polyvinyl alcohol (PVA, 1750 ± 50) was purchased from Shanghai Yuanye Biotechnology Co., Ltd. Polyvinylpyrrolidone (PVP, K-60) was purchased from Beijing Kulaibo Technology Co., Ltd. Matrine (MAT) was purchased from China National Pharmaceutical Group Chemical Reagent Co., Ltd. The reverse transcription kit and PCR reagents were purchased from Aibimeng Biotechnology Co., Ltd. MAT ointment was purchased from Youkang Lipang Pharmaceutical Co., Ltd. Sodium periodate (NaIO_4_), sodium bicarbonate (NaHCO_3_), dinitrochlorobenzene (DNCB), trypsin EDTA digestion solution, PBS buffer, DMSO, propidium iodide (PI), calcein AM, trypan blue, simulated body fluid (SBF), CCK-8 cell proliferation and cytotoxicity test kit, calcifein AM/PI double staining kit, hematoxylin eosin/HE staining kit, Masson’s Trichrome Stain Kit, and phosphate buffer solution (PBS) were provided by Beijing Solaybao Biotechnology Co., Ltd. Human biological immortality keratinocyte (HACAT) was purchased from Procell Life Technology Co., Ltd. All animal experiments were conducted in accordance with the National Research Council Guide for the Care and Use of Laboratory Animals.

### Construction of self-adhesive swelling microneedles

The self-adhesive swelling microneedles (SDSMNs) were composed of swelling needle tips and adhesive substrate. They were constructed using a two-step casting method that employed a PDMS micro-mold (20 mm × 20 mm, 35 × 35 array, pyramid geometry, 600 μm of needle tips).

#### Preparation of swelling microneedle tips

A PVA solution with different concentrations was mixed with PVP solution at various volume ratios. The PVA/PVP solution (1 mL) was cast into the needle cavities of PDMS mold and centrifuged at 4000 r/min for 5 min. The solution residue was carefully removed and then held at 60 ℃ for 1 h to obtain the microneedle tips.

#### Preparation of adhesive substrate

The adhesive substrate was prepared with dopamine and gelatin via an oxidation reaction. First, gelatin (G-A or G-B) was dissolved in DI water at 55 ℃ under stirring condition at final concentration of 31.25% (m/v) and supplemented with 2.5% (v/v) glycerol. As in warm status, dopamine hydrochloride was added at a final concentration of 6.25% (m/v). Serial concentrations of NaIO_4_ or NaHCO_3_ solution (2–5%, m/v) was then, respectively, added at a volume ratio of 1:4 gelatin solution that was dispersed evenly. The obtained pre-solution was cast onto the microneedle tips, cooled down at 4 ℃ for quick gelation, then left to dry in the dark at room temperature for 36 h. The substrate was oxidized using NaIO_4_ or NaHCO_3_ during drying process, designated as PG or BG, respectively.

### Morphological observation

The SDSMNs were photographed then cut into pieces that were sputtered with gold then observed with scanning electron microscope (JSM-840, Bruker Company, Switzerland). The needles of SDSMNs were labeled with DiI. The needle array was observed using an inverted fluorescent microscope. Moreover, the dried substrates with various oxidation crosslinks were immersed in simulated body fluid (SBF) for 48 h before freezing at −80 ℃. Samples were lyophilized for SEM observation.

### Rheological test of SDSMNs substrate

To assess the effect of oxidation crosslink on viscoelasticity of substrate, rheological performances were examined using HR 10 Rheometer (TA Instruments, U.S.). Two (2) mL pre-solution of PG or BG with different concentrations of oxidizing agent was cooled down and maintained at 4 ℃ for gelation in 6-well plate and then held for 3 h in the dark before testing. The gels were carefully removed and placed onto the platform with a gap of 1200 μm between the 40 mm diameter parallel plates. Samples were left for 10 min at 25 ℃ to equilibrate before measurements. The angular frequency of 1 rad/s was set to perform a strain sweep test in the range of 0.01−100% strain. The frequency sweep tests were employed from 0.1 to 100 rad/s under a constant strain of 1%.

### Tensile strength test of SDSMNs substrate

Tensile strength of SDSMNs substrate was tested on a universal testing machine (CTM2050S) following ASTM F2258. A pre-solution of substrate (1.2 mL) was added to a mold (20 mm × 40 mm) and dried in the dark for 48 h. The dried substrate was clamped at both ends with one end fixed and the other end moved at 4 mm/min until a fracture occurred. The tensile strength was measured using a 1000 N gravity sensor, calculated by dividing the maximum load by the cross-section area of the substrate film.

### Adhesion capacity of SDSMNs substrate

The adhesion capacity of SDSMNs substrate was evaluated using porcine skin using a 90-degree peel test in accordance with ASTM D6862. Fresh, shaved porcine skin obtained from the flanks of a pig and the dermal surface were cleaned with isopropyl alcohol and gauze. The subcutaneous fat layer was removed using a scalpel. Skin samples were sealed and stored at -20℃. The skin tissue was thawed and sized into 25 mm × 40 mm. The dermis side of skin was glued onto a piece of slide. One end of the SDSMNs substrate (20 mm × 50 mm) was applied to the epidermis side of porcine skin with an overlapping area of 20 mm × 40 mm, while the free end was clamped onto the fixture. A 100 g weight was pressed onto the overlapping part for 2 min. The peel strength of the substrate was measured at a constant speed of 10 mm/min using a 100 N gravity sensor at room temperature, calculated by dividing the maximum load by the width of the adhesion area.

A piece of substrate film (10 mm × 25 mm) was applied onto the surface of finger or wrist joints and pressed for 3 min. When the joints were bent repeatedly to 90-degree, photos were taken to evaluate the adhesion capacity and stability.

### Swelling properties of SDSMNs

The microneedle prepared with PVA: PVP = 3:1 (20% HPVA and 30% PVP) and PG (4%) was taken, and the mass M_0_ of the initial microneedle material recorded. The microneedle was immersed in SBF at 37 °C and taken out at 10, 20, 60, 120, 360, or 480 min. The excess water on the surface was removed using filter paper. The microneedle was then weighed and the mass recorded as Mt. The swelling rate of the microneedle was expressed as (Mt−M_0_)/M_0_ × 100%. Each group was tested three times.

### Drug release of matrine-loaded SDSMNs (MAT/SDSMNs)

For preparation of MAT-loaded SDSMNs, MAT was evenly pre-mixed with PVA/PVP solution (1 mL) at a final concentration of 40 mg/mL then cast into the needle cavities during construction. The MAT-SDSMNs were immersed into PBS (pH 7.4) and incubated at 37 ℃ while stirring. A 1 mL solution was sampled at 1, 3, 6, 12, 18, 24, and 36 h, respectively, and supplemented with same amount of fresh PBS. The content of MAT was determined by liquid chromatography (HPLC, Agilent 1260, USA) equipped with a C18 column (150 mm × 3.9 mm) at a UV absorption wavelength of 220 nm. The mobile phase consisted of water (with 0.1% formic acid) and acetonitrile (50:50 v/v) at flow rate of 1 mL/min. A blank SDSMNs was used as a control.

In addition, Franz cell (infiltration area is 1.76 cm^2^ and the volume of receiving cell is 13 mL) was applied to determine the transdermal drug delivery of MAT-SDSMNs. Simulated skin was constructed using 2% agarose solution with a thickness of 3 mm (Shi et al. 2022). During the experiment, the simulated skin was fixed onto a diffusion cell with one side was in close contact with the receiving solution (PBS, pH = 7.4). The apparatus was stirred (250 r/min) and equilibrated at 32 ℃ for 15 min. MAT-SDSMNs were inserted into the upper surface of the skin, samples were taken at a predetermined time interval to determine the MAT concentration. Fresh PBS supplemented immediately. Cumulative active ingredient penetration (Q, μg/cm^2^) through the simulated skin was calculated using the following equation:

$$ {\text{Q}}\, = \,{\text{VrCt}}\, + \mathop \sum \limits_{i = 0}^{t - 1} {\text{VsCi,}} $$

where Ct is the drug concentration of the receiver solution at each sampling time, Ci is the drug concentration of the *i*th sample, and Vr and Vs are the volumes of the receiver solution and the sample, respectively. Data were expressed as the cumulative drug permeation per unit of simulated skin surface area.

### Antibacterial activity of SDSMNs substrate

This antibacterial activities of SDSMNs substrate were tested using *S. aureus* (ATCC29213) and *E. coli* (ATCC25922). Freshly prepared SDSMNs substrate hydrogels were immersed with LB media and incubated at 37 ℃ for 24 h. The extraction was then filtered through 0.22 μm sterile filter. The extract was diluted with LB media to obtain 0, 5, 10, 15, 20, 30, and 40 mg/mL dilutions, which were added into 96-well plate with 90 μL per well that was supplemented with a 10 μL bacterium suspension to get a final bacterial density of 1 × 10^5^ CFU/mL. The plate was incubated at 37 ℃ for 20 h and the absorbance measured at 620 nm using an ELX800UV microplate reader (Bio-Tek, U.S.) for minimum inhibitory concentration (MIC) determination.

In addition, the antibacterial activity of SDSMNs substrate was also verified by Kirby-Bauer (KB) test. The filter paper and SDSMNs hydrogels were sized into round sheets, irradiated under the UV lamp for 12 h. Bacterium were second activated and cultured until reaching the plateau stage. The obtained bacterium suspension was diluted with LB medium for 10^0^-, 10^1^-, 10^2^-, 10^3^-, 10^4^-, and 10^5^-fold, and dispersed onto agar plates. The filter paper and SDSMNs substrate films were placed onto the plate, cultured at 37 ℃ for 16 h, then the inhibition zones were observed and photographed. The filter paper sheets soaked with 0.7% normal saline served as the negative control.

### In vitro assessment of therapeutic efficiency

#### Establishment of eczema cell model

HACAT cells were inoculated into 6-well plates at a density of 1 × 10^5^ cells per well and cultured for 6 h. 100 μL IL-17 solution (10, 20 ng/mL) was added to each well and the HACAT cells cultured for 24 h. The cells without IL-17 treatment were used as a control.

##### qRT-PCR detection of IL-17R expression

Total RNA from HACATs was isolated using RNA Extraction Reagent (Servicebio®). cDNA was generated using the Servicebio® RT First Strand cDNA Synthesis Kit (Servicebio®). Quantitative qRT-PCR was performed using the SYBR Green qPCR Master Mix (High ROX) and an ABI Stepone plus Sequence Detection System. PCR cycling conditions were as follows: denaturation for 10 min at 95 °C, followed by 40 cycles of denaturation (95 °C for 15 s), annealing (60 °C for 20 s), and extension (72 °C for 10 s). GAPDH expression was used to normalize data using the ΔCt method. Fold changes in relative gene expression of IL-17R were identified using the 2^−ΔΔCt^ method. Primer sequences used in this study are in Table S1.

##### Cytokine detection

Typical eczema proinflammatory cytokines IL-6 and TNF- α and IL-1 β were selected for characterization. Briefly, the supernatants of cell culture were collected by centrifugation at 5000 r/min for 15 min. ELISA kits were used to detect the level of cytokines.

#### Therapeutic efficiency assay

After stimulation with 10 ng/mL IL-17 for 24 h, the culture medium was discarded and replenished with MAT at 250 or 500 μg/mL with or without IL-17, then further cultured for 24 h. HACAT cell without IL-17 stimulation served as the negative control. qRT-PCR and cytokine detection were carried out to evaluate therapeutic efficiencies.

##### Cell migration Assay

HACATs were inoculated in dishes and cultured in 5% CO_2_ at 37 ℃. After 24 h, IL-17 (10 ng/mL) and MAT + IL-17 CL (250 μg/mL MAT and 10 ng/mL IL-17) were added to the culture medium, respectively, and confluent cell layers scratched with 200 μL pipette tip. Images were taken with an inverted phase contrast microscope at 0, 12, 24, and 36 h.

### In vivo assessment of therapeutic efficiency

An eczema animal model was developed following a reference method (Kang et al. 2011). SD rats (7 weeks old, 200 g, female) were used in this experiment. The rats were adapted for 7 days then their dorsal skin were shaved and depilated with hair removal cream. 24 h later, 100 μL DNCB in acetone (5%) was applied on the bare skin for eczema induction. While on days 7, 11, 15, and 19, the treated skin area was challenged with 100 μL DNCB (2%) to maintain eczema symptoms. The untreated rats were served as the control. The rats were randomly grouped into four groups (*n* = 5) 24 h after challenging—MAT ointment, subcutaneous injection of MAT solution, MAT-MN, and control group (equivalent MAT concentration of 0.5 mg/mL). Treatments were performed on days 8, 12, 16, and 20. Rats were killed and the skin scored according to severity scoring atopic dermatitis (SCORAD) scoring scale (supplementary materials) then histological analysis was performed. Blood samples were also collected for cytokine analysis.

### Statistical analysis

All experiments were independently repeated at least three times. Data was plotted using mean ± standard deviation (SD). Statistical analysis was conducted using Graphpad Prism 8.3.0 software. Differences in data were analyzed using one-way or two-way analysis of variance (ANOVA). Levels of significant were as follows—* equals (*P* < 0.05), ** equals (*P* < 0.01), and *** equals (*P* < 0.001), while ns represented no significant difference.

## Results and discussion

A self-adhesive swelling microneedle (SDSMNs) with adhesive substrate and swelling needle tips was designed and constructed for use in this study (Fig. 1). The adhesive substrate enabled adhesion of the microneedle patch on skin surface via self-administration, avoiding translocation or detachment, while the swelling needle tip released loaded MAT in a controlled fashion through absorbing interstitial fluid.

### Preparation and characterization of adhesive substrate

Gelatin and dopamine have been widely used in the development of adhesive materials (Gowda et al. 2020; Montazerian et al. 2021). Under oxidation, an activated catechol group of dopamine generates a quinone that is highly reactive with thiol and amine groups via Michael-type addition or Schiff-base formation reactions, along with the hydrogen bond and π–π stacking. This results in the crosslink with gelatin and adhesion on tissue interface.

**Fig. 2:**
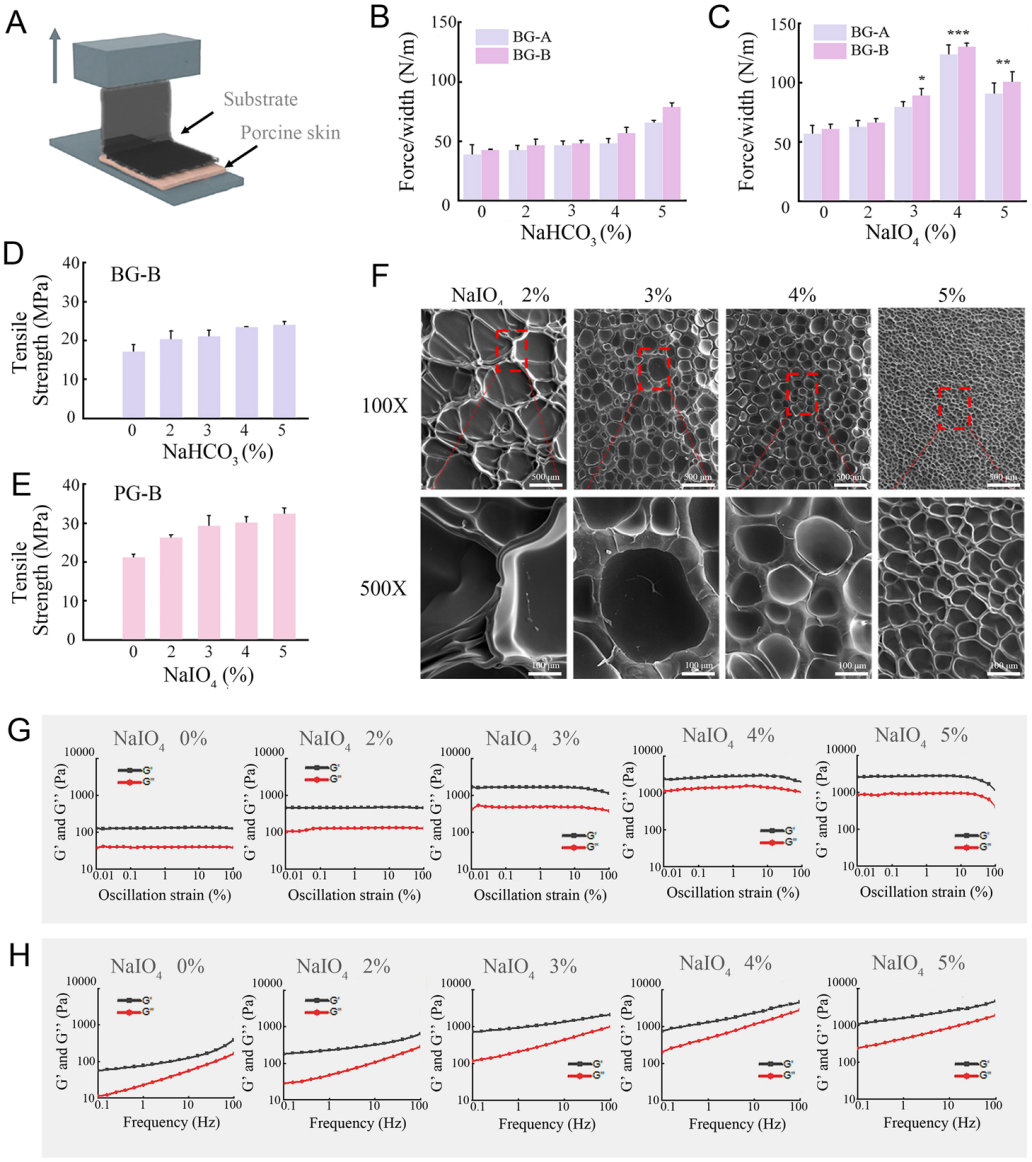
Characterization of microneedle adhesive substrates. **A** Schematic image of 90° peeling test for adhesion evaluation using porcine skin. The adhesion strength as a function of NaHCO_3_ (**B**) or NaIO_4_ (**C**) concentration. Tensile strength of substrate derived from gelatin B as function of NaHCO_3_ (**D**) or NaIO_4_ (**E**) concentration. **F** SEM images of PG-B substrates with different degrees of oxidation. Rheological test in term of strain sweep (**G**) and frequency sweep (**H**) as function of NaIO_4_ concentration. Data depict mean ± standard deviation, * *P* < 0.05, ** *P* < 0.01, *** *P* < 0.001 (colour figure online)

An adhesive substrate was prepared by blending gelatine (G-A or G-B) and dopamine under two oxidative conditions, weak oxidation in NaHCO_3_ solution termed as (BG-A or BG-B) and strong oxidation in NaIO_4_ termed as (PG-A or PG-B). The adhesive capacity of adhesive substrates in hydrogel form was assessed using a 90-degree peeling test (Fig. 2A). As shown in Fig. 2B and C, substrates derived from gelatin B always generated stronger adhesion than gelatin A, presumably because of the higher molecular weight of B type gelatin that fabricates a stronger crosslink network and mechanical property (Gómez-Guillén et al. 2009). In addition, PG displayed stronger adhesion than BG. As for BG, adhesive force slowly increased with NaHCO_3_ concentration and reached a maximum of 65.78 N/m at 5%. In case of PG, the adhesive force peaked at 4% NaIO_4_ of 129.8 N/m and decreased at a higher concentration. The results suggest adhesive strength is closely related to oxidation. On the other hand, the catechol group of dopamine undergoes oxidative polymerization forming a chemical cross-linked network with gelatin molecular chain through Michael addition and Schiff-base reaction. Whereas, the unoxidized catechol groups could bind to active groups (e.g., NH_2_, SH, OH, and COOH) on tissue interface through covalent interactions or non-covalent interactions (hydrogen bonding or electrostatic interactions). Tissue adhesion has a synergetic effect of cross-linked network mechanical properties and interface adhesion. Moderate oxidation promoted covalent crosslinking between dopamine and gelatin, enhancing the network mechanical strength, while the unoxidized catechol groups provided interface adhesion. However, excessive oxidation, such as 5% NaIO_4_, would consume the free catechol groups of dopamine, reducing the interface adhesion, which resulted in the decline of tissue adhesion. Considering the higher bonding strength of PG-B substrate, PG-B oxidized by 4% NaIO_4_ was used for subsequent experiments.

Tensile strength of dry substrate films derived from gelatin B in different oxidation process was detected. As shown in Fig. 2D and E, the tensile strength of substrate was positively associated with an increasing concentration of NaHCO_3_ or NaIO_4._ PG-B exhibited higher strength than BG-B substrate, demonstrating a higher degree oxidation of PG-B, which formed a stronger cross-linked network that could withstand higher tensile strength. In addition, the tensile strength of PG-B at 4% NaIO_4_ was 30.07 MPa, lower than 5% NaIO_4_, suggesting there were still free catechol groups in the PG-B network that could be used for interface adhesion. The result is supportive and consistent with the highest tissue adhesion strength shown by PG-B in Fig. 2C. SEM image of PG-B substrate displayed porous structure of the network. The pore size reduced along with NaIO_4_ concentration (Fig. 2F), reflecting the increased degree of cross-linked network. Moreover, more cross-linked network (e.g., 5% NaIO_4_ treatment) resulted in increased stiffness of the substrate that would generate lower flexibility to attach to irregular tissue interfaces, compromising its adhesion capacity. These results agree with the adhesion and tensile strength data.

Because the degree of oxidation affects the cross-linked network of PG-B substrate, the mechanical property of PG-B hydrogel was also studied by rheological assay. When the oscillation strain varied from 0.01 to 100%, the storage modulus (G′) was higher than the loss modulus (G″), which did not show obvious change (Fig. 2G). This indicated PG-B hydrogel was stable and the elasticity was independent upon the shear frequency within the sweeping range, demonstrated to be the linear viscoelastic region. Moreover, the value of G′ increased with NaIO_4_ concentration, reflecting the cross-linked network was more stable. As for frequency sweep, the strain was fixed at 1%. A similar phenomena appeared again except that both G′ and G″ gradually increased with the rising NaIO_4_ concentration (Fig. 2H), indicating the degree of oxidation and internal crosslinking of hydrogels were strengthened. These results are also consistent with tensile strength results.

### Construction and characterization of matrine-loaded SDSMNs

As a transdermal drug delivery formulation, especially for eczema and other skin diseases, microneedles must ensure sufficient mechanical strength to puncture the thickening epidermis. The needle tips were designed to be mechanically strong and swellable in the presence of interstitial fluid. SDSMNs were constructed using a two-step casting method, giving rise to a heterogeneous appearance of black substrate and semi-apparent needles (Supplementary Fig. S1Aa). An ordered array of needles could be seen in SEM images, each pyramid needle with a bottom edge length of 300 μm and a height of 500 μm (Supplementary Fig. S1Ab). A clear boundary was observed between the needles and substrate (Supplementary Fig. S1Ac), suggesting the drug could be well concentrated into the needles, hence, improving the transdermal delivery efficiency afterward.

The China Food and Drug Administration (FDA) approved polymers PVA and PVP were used for screening and fabrication of the needle tip. In a previous study, we developed swelling needles by simply adjusting the alcoholysis degree of PVA (Shi et al. 2022). When blending with PVP, known as a superior mechanical property, the PVA/PVP texture is expected to be mechanically enhanced. As shown in Supplementary Fig. S1B, the axial-fracture force per needle increased with PVA concentration or PVA/PVP volume ratio. The highest mechanical strength of the microneedle was obtained at volume ratio of 20% PVA–30% PVP = 3:1, so each needle could withstand a maximum force of 0.63 N. The swelling properties of SDSMNs prepared with the screened needles and PG-B substrate were characterized with a swelling rate of 530%, after 8-h incubation in simulated body fluid (Supplementary Figs. S1C, S2). Skin insertion capacity of the microneedle was assayed using fresh mice skin or simulated skin, both of which showed an insertion rate of 97% (Supplementary Fig. S3).

As expected, MAT/SDSMNs displayed a sustained release of MAT in vitro, reaching 80% at 12 h and a maximum of 90.3% at 24 h (Supplementary Fig. S1D). In addition, simulated skin was mounted upon a Franz cell to assess transdermal delivery efficiency. A dissolving microneedle (DMN) formulated with PVP served as a control (Supplementary Fig. S1E). The cumulant amount of drug released by swelling microneedle (SMN) was significantly larger than DMN, which was 1.14-fold at 20 h (Supplementary Fig. S1F). This was mainly attributed to the cross-linked network of SMN, which obviously inhibited the diffusion of small molecule drugs in the needle matrix extending the release duration.

**Fig. 3:**
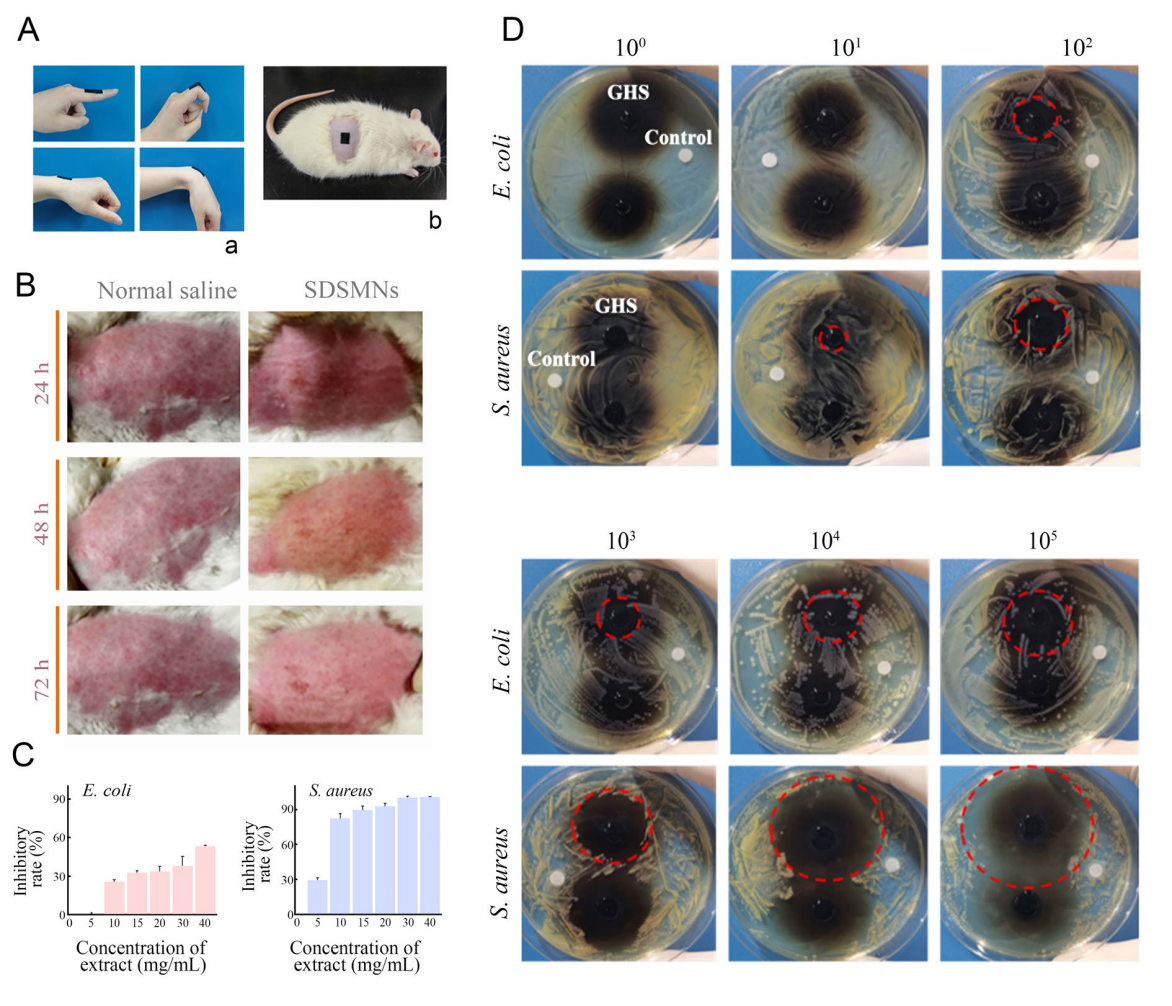
**A** Representative images of adhesion of SDSMNs on (**a**) different joints skin surface under repeated bending movements or (**b**) r depilated rat skin. **B** Skin irritation of SDSMNs on New Zealand rabbit skin after adhesion for 24, 48, and 72 h. **C** The antibacterial rate of PG-B against *E. coli* and *S. aureus.* **D** The antibacterial activities of PG-B against the growth of *E. coli* and *S. aureus* of different concentrations using inhibition zone assessment (colour figure online)

Eczema occurs in various parts of the skin surface, including skin that covers large and small joints. The adhesion performance of SDSMNs was tested by application on the surface of finger or wrist joints (Fig. 3Aa). The adhesion could withstand repeated 90° bending movements without any striping or shedding. SDSMNs could be also readily adhered on the depilated rat skin (Fig. 3Ab). The results verified that SDSMNs could generate universal and efficient adhesion on skin surface. As noted, long duration of attachment on skin requires biosafety of SDSMNs during the attachment period, which was evaluated via hemolysis, cytotoxicity, skin irritation, and antibacterial activity assessments. Hemolysis rates of PG-B substrate extract under all tested concentrations were less than 5% (Supplementary Fig. S4). PG-B was non-cytotoxic toward HACAT or L929 when the extract concentration was no more than 1 mg/mL, which was also compatible at 5 mg/mL against HACAT cells (Supplementary Fig. S5). PVA/PVP needles had no toxicity toward the cells at all test concentrations (Supplementary Fig. S6). SDSMNs also showed no skin irritation that was comparable to normal saline after different period of attachment on depilated rabbit skin (Fig. 3B). Gram-positive bacteria *S. aureus* is a typical strain found in eczema and Gram-negative bacteria *E. coli* is a strain closely related to infection of common skin diseases. PG-B hydrogel prepared with 4% NaIO_4_ was selected to test its antibacterial activity. The MIC of PG-B was first measured by incubating bacterium with PG-B hydrogel extract, which was 10 mg/mL and 5 mg/mL against *E. coli* and *S. aureus*, respectively, showing a stronger antibacterial effect against *S. aureus* (Fig. 3C). When the concentration of PG-B was higher than 20 mg/mL, the inhibitory rate was more than 90% toward *S. aureus*, which was desirable to avoid any infection risk in the area of eczema. Moreover, in a Kirby–Bauer (KB) test, round sheets of PG-B film were incubated with bacteria for 16 h before visualizing the survival of both *S. aureus* and *E. coli* by culturing them on the agar plate. Both *E. coli* and *S. aureus* showed bacteriostatic circles on agar plates, and larger circles appeared against *S. aureus*, which again confirmed its stronger antibacterial activity (Fig. 3D).

### In vitro therapeutic efficiency assessment

**Fig. 4:**
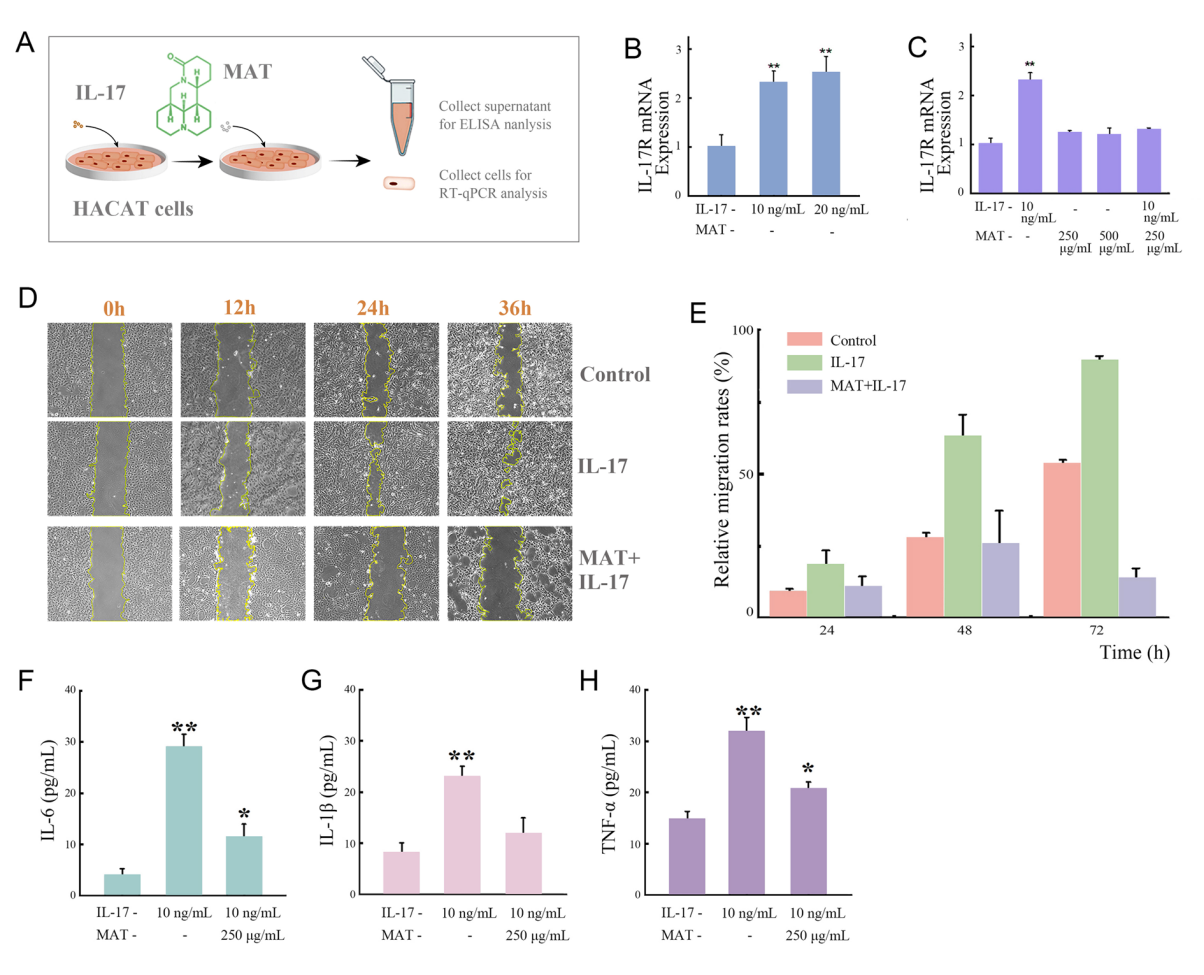
The therapeutic effect of MAT on eczema cell models. **A** Schematic of protocol. **B** The expression of IL-17R mRNA in HACAT cells induced with IL-17 (10, 20 ng/mL), the cells without any treatment were taken as negative control. **C** After stimulation with 10 ng/mL IL-17, the effect of MAT (250, 500 μg/mL) on the expression of IL-17R mRNA with or without IL-17 continuous treatment. **D** Confluent monolayers of HACAT cells were scratched with a pipette tip and incubated with 10 ng/mL IL-17 or MAT + IL-17 (250 μg/mL MAT and 10 ng/mL IL-17) for 0, 12, 24, and 36 h, respectively. The untreated group served as a negative control (scale bar = 200 μm). **E** The relative migration rates of HACAT cells. The effect of MAT (250 μg/mL) on the levels of IL-6 (**F**), IL-1β (**G**) and TNF-α (**H**) secreted by HACAT cells after treatment with IL-17 (10 ng/mL). Data depicted as mean ± standard deviation, * *P* < 0.05, ** *P* < 0.01, *** *P* < 0.001 (colour figure online)

Cytotoxicity of MAT was first detected to determine the safe dose in application. It is not toxic to HACAT or L929 cells with a concentration no more than 0.5 mg/mL (Supplementary Fig. S7). IL-17 is mainly produced by Th17 cells, playing a major role in the recruitment of inflammatory cells into atopic dermatitis, such as eczema (Heo et al. 2015; Hofmann et al. 2021). IL-17 was used to induce eczema-like inflammation reactions on HACAT cells, which was used as a model to assess therapeutic efficiency of MAT (Fig. 4A). As shown in Fig. 4B, mRNA expression of IL-17R, receptor of IL-17 in cells, was significantly upregulated with treatment of 10 ng/mL IL-17, while an even larger dose (20 ng/mL) would not lead to further increase. IL-17 is associated with the occurrence and development of various immune diseases and inflammatory conditions by binding to the IL-17 receptor expressed on keratinocytes. This stimulates the proliferation and activation of keratinocytes, causing signal transduction and the induction of pro-inflammatory factors, which mediate defense responses. (Herjan et al. 2018). As expected, the migration of HACAT cells was promoted in presence of IL-17 and enhanced along with treatment time (Fig. 4D, E). Inflammatory cytokines including IL-6, IL-1β and TNF-α in cell culture supernatant were detected (Fig. 4F–H). The levels of all cytokines were significantly higher than the negative control, which was consistent with the reference (Homey et al. 2000). Results verified the eczema cell model was successfully established.

To test the therapeutic effect of MAT on eczema, 10 ng/mL IL-17 was used to induce HACAT cells. After 24 h, MAT (no more than 500 μg/mL) was added with or without continuous stimulation of IL-17. It was found that 250 μg/mL MAT significantly reduced the mRNA expression of IL-17R, even under co-stimulation with 10 ng/mL IL-17 (Fig. 4C). The enhanced cell migration was inhibited and returned to the initial state for MAT + IL-17 group (Fig. 4D, E). The promoted secretion of cytokines by HACAT cell was significantly inhibited after treatment with 250 μg/mL MAT (Fig. 4F–H). These results indicated MAT could significantly alleviate the inflammatory responses mediated by IL-17 in HACAT cells.

### In vivo therapeutic efficiency assessment

**Fig. 5:**
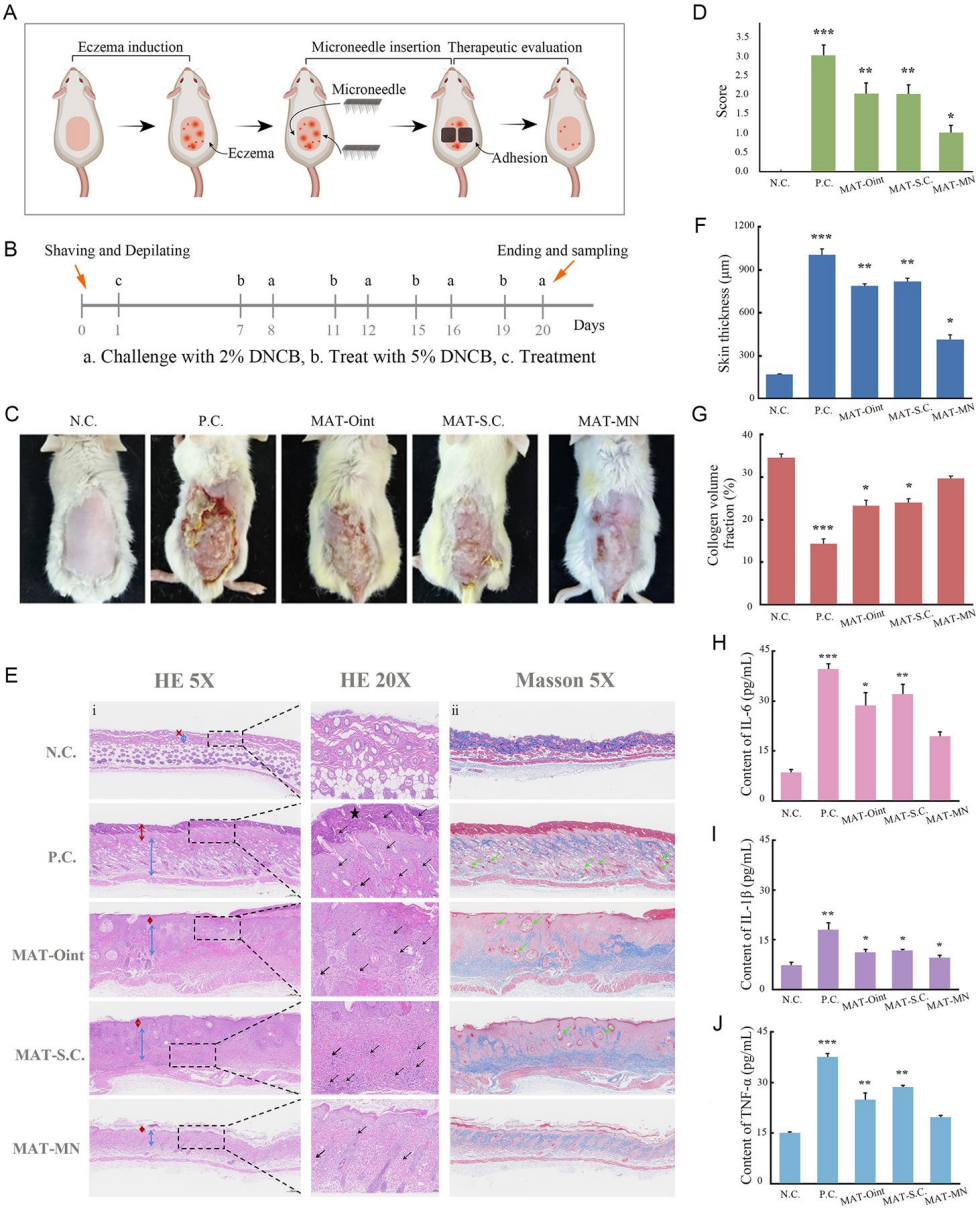
Therapeutic efficiencies of MAT-SDSMNs on eczema rat model. **A** Schematic of eczema induction and treatment using MAT-SDSMNs on rat model. **B** Timeline of eczema induction and treatment on eczema rat model. **C** The appearance of the skin surface of rats at the end of treatment with MAT-Ointment (MAT-Oint), MAT subcutaneous injection (MAT-SC) or MAT-SDSMNs (MAT-MNs) (n = 5). The rats without treatment served as the negative control (NC). Ones treated with DNCB were the positive control (PC). **D** Score of eczema symptoms using the SCORAD index. **E** Histological section analysis of skin tissue after various treatments. (i) H&E staining, (ii) Masson staining. The red double arrow represents the epidermis, the blue double arrow represents the dermis, the black ★ represents the parakeratosis, the black arrow represents the inflammatory cells, and the green arrows represents dilated blood vessels. **F** The skin thickness of rat after various treatments. **G** The collagen deposition fraction in rat dermis after various treatments. The levels of IL-6 (**H**), IL-1β (**I**) and TNF-α (**J**) measured in plasma after various treatments. Data depicted as mean ± standard deviation, * *P* < 0.05, ** *P* < 0.01, *** *P* < 0.001 (colour figure online)

Our eczema model was induced using DNCB stimulation on rat skin (Fig. 5A, B). The rats were randomly divided into 5 groups—negative control (normal skin), positive control (without treatment), treated with MAT-Ointment (MAT-Oint), subcutaneous injection of MAT (MAT SC), and MAT-MN. As shown in Fig. 5C, the severity of PC scored the highest 3 points, characterized as obvious erythema, edema, exudation, and lichenification with skin ulceration. After treatment, symptoms were alleviated to different degrees. Edema, exudation, excoriation, and lichenification were still obvious in the MAT-Oint and MAT SC groups but were not observed in the MAT-MN group leaving only erythema after ulceration repair with a SCORAD score of 1.0 (Fig. 5D).

To further evaluate the therapeutic efficiency, histological sections of rat lesion skin on day 20 were prepared for HE and Masson staining (Fig. 5E). As shown in Fig. 5Ei, larger number of infiltrated inflammatory cells (black arrows) were observed in the PC group, especially in the epidermal layer along with parakeratosis (black asterisk), with either the epidermic or dermis significantly thickening, which are both typical signs of eczema. After treatment with MAT formulations, the inflammation was dramatically alleviated. The skin thickness of MAT-MN was reduced to 40% of the PC group and to 50% of the MAT-Oint or MAT-SC groups (Fig. 5F). The Masson staining results showed collagen fibers in the PC group were in disorder arrangement and content significantly reduced compared with the treatment groups (Fig. 5Eii). Moreover, the blood vessels in lower dermis were dilated and congested with thickened walls and incomplete structure (green arrows), resulted into exudation. The phenomena were also found in the MAT-Oint and MAT-SC groups. As for MAT-MN, the collagen fibers significantly increased and were more orderly arranged in the dermis than in the other groups. The collagen deposition fraction was 29.43% (Fig. 5G), which is close to normal skin. In addition, dilation and congestion of blood vessels disappeared, as is consistent with no edema for the MAT-MN treated group Fig. 5C. Levels of pro-inflammatory cytokine IL-6, IL-1 β and TNF- α in serum were detected. Rats treated with MAT-MN displayed the lowest levels of inflammatory cytokines (Fig. 5H–J), probably due to the controlled release of MAT in skin tissue compared with ointment or subcutaneous injection. Results agree well with the histological assay, indicating MAT-MN could improve the therapeutic efficiency of eczema treatments by regulating the inflammatory microenvironment of eczema site via reducing inflammation reactions.

## Conclusion

Eczema is a common chronic inflammatory skin disease that is difficult to treat. In this study, a matrine-loaded self-adhesive swelling microneedle (MAT-SDSMNs) was developed via a simple method. MAT-SDSMNs could adhere onto the eczema surface after insertion into skin and avoid detachment, while the swelling needles could release the MAT in a controlled manner. MAT-SDSMNs was biocompatible and safe toward skin tissue. Additionally the inherent antibacterial activity could prevent infection during application. Compared with conventional formulations, MAT-SDSMNs exhibited better in vivo therapeutic efficiency through alleviating local inflammation and significantly relive eczema symptoms, providing a simple, self-administered, and sustainable strategy for eczema treatment.

### Supplementary Information

Below is the link to the electronic supplementary material.

Supplementary file1 (DOCX 2437 KB)

## Data Availability

The data used to support the findings of this study are available from the corresponding author upon request.
