# Supplementary material for: Matrine-loaded self-adhesive swelling microneedle for inflammation regulation to improve eczema treatment
Source: Mar Life Sci Technol. 2024 Jun 26;6(3):535–46. doi: 10.1007/s42995-024-00235-z (PMC11358580; doi:10.1007/s42995-024-00235-z)
Supplement: Supplementary file 1 — Supplementary file1 (DOCX 2437 KB) [file 42995_2024_235_MOESM1_ESM.docx]

**Supplementary materials**

**Cytotoxicity assay**

CCK-8 assay

L929 and HACAT cells used in this experiment were purchased from the Cell Bank of Chinese Academy of Sciences. The cells were harvested at logarithmic growth period, centrifuged and resuspended with culture medium (containing 10% FBS) at density of 5×10^4^ cells/mL. 100 μL/well cell suspension was inoculated into 96-well plates and cultured at 5% CO_2_ concentration and 37℃ for 12 h. The medium was discarded and replaced with extract of microneedle substrate or needle tips (0.01, 0.1, 1, 5, 10 mg/mL), or MAT solution (0.01, 0.1, 0.5, 1, 10 mg/mL). The blank control group was added with 100 μL medium and cultured for 24 h, 48 h and 72 h. The 96-well plates incubated for a specific time were taken out of the incubator and 5 μL CCK-8 solution was added into each well for incubation at 37°C for 2 h in the dark condition. The absorbance values (OD) of each group were determined by microplate reader at 450 nm.

Live/dead staining assay

For the morphology observation, the cells were stained with 5 uL calcein-AM and Propidium Iodide for incubation at 37°C for 15 to 20 minutes and each well was washed with PBS for three times to ensure that the residual dye in the medium was removed. The cells in the well plates were soaking with 100 μL of PBS and the cells were observed by fluorescent inverted microscope (Nikon, Ts2R, NIS-Elements). Excitation light was set at 495 nm with the fluorescence emission was recorded between 510 and 530 nm, and 536 nm with the fluorescence emission was recorded around 617 nm.

**Hemolysis assay**

The cytocompatibility of microneedle substrate (PG-B) was verified by hemolysis of red blood cells. Fresh 1 mL of New Zealand white rabbit blood was taken and placed in heparin sodium anticoagulation tube to prevent blood agglutination. PG-B substrate was incubated in normal saline (0.7%) at 37℃ for 24 h, the extract supernatant was obtained and diluted to gradient concentrations. 20 μL blood was added to 1 mL sample extract, deionized water (positive control) and normal saline (negative control), respectively. The mixture was shaken in water bath at 37℃ for 1 h, centrifuged at 2000 rpm for 5 min, and the absorbance value of supernatant was detected at 545 nm using a microplate analyzer. Hemolysis rate (HR, %) was calculated by the following equation:

HR%=$\frac{\text{OD}\text{s}\text{-OD}\text{nc}}{\text{ODpc-OD}\text{nc}}$×100

where OD_s_, OD_nc_, and OD_pc_ were the absorbencies of the tested sample, negative control, and positive control, respectively.

**Mechanical property of MNs**

The mechanical properties of the MNs arrays (10 × 10 needles) were evaluated using universal testing machine. The MNs patch was placed on the test plate with tips facing up and underwent compression in vertical direction at a speed of 0.1 mm/min using a 100 N gravity sensor.

***In vitro* skin insertion assay**

The 10 ×10 array microneedles were vertically inserted into freshly depilated mice skin, and the inserted microneedles were pressed with 15 N force for 1 minute. The skin surface was stained with Trypan blue for 30 minutes. After trypan blue was removed with 75% ethanol, the number of stained pinholes was observed and recorded. The insertion capacity was also tested using simulated skin (2% agarose solution with a thickness of 3 mm) and DiI labeled microneedle.

**Skin irritation assay**

New Zealand white rabbit (3 kg) was used for skin irritation assay in accordance with ISO 10993-10. The rabbits were allowed to adapted for 7 days before test. After being anaesthetized, the hair on both sides along the spine was shaved carefully, leaving bare skin areas of 5 cm × 10 cm. 24 h later, SDSMNs patches were applied on one side of spine, while the other side was treated with saline absorbed cotton swab as negative control. The skin contact was maintained for at least 4 h before the SDSMNs were taken off and cleaned with saline. The skin responses were recorded at 24, 48 and 72 h.

**Severity Scoring Atopic Dermatitis (SCORAD) score**

The main results of the study were the assessments of the area under the curves of the total SCORAD score and of the use of treatment during the 7-day treatment period as previous report (Wang et al. 2022). The scores were divided into four grades, 0 point: normal skin. 1 point: mild erythema, edema, no exudation. 2 points: erythema, edema with a small amount of exudation, accompanied by scarring. 3 points: obvious erythema, edema and exudation, with skin ulceration.

**
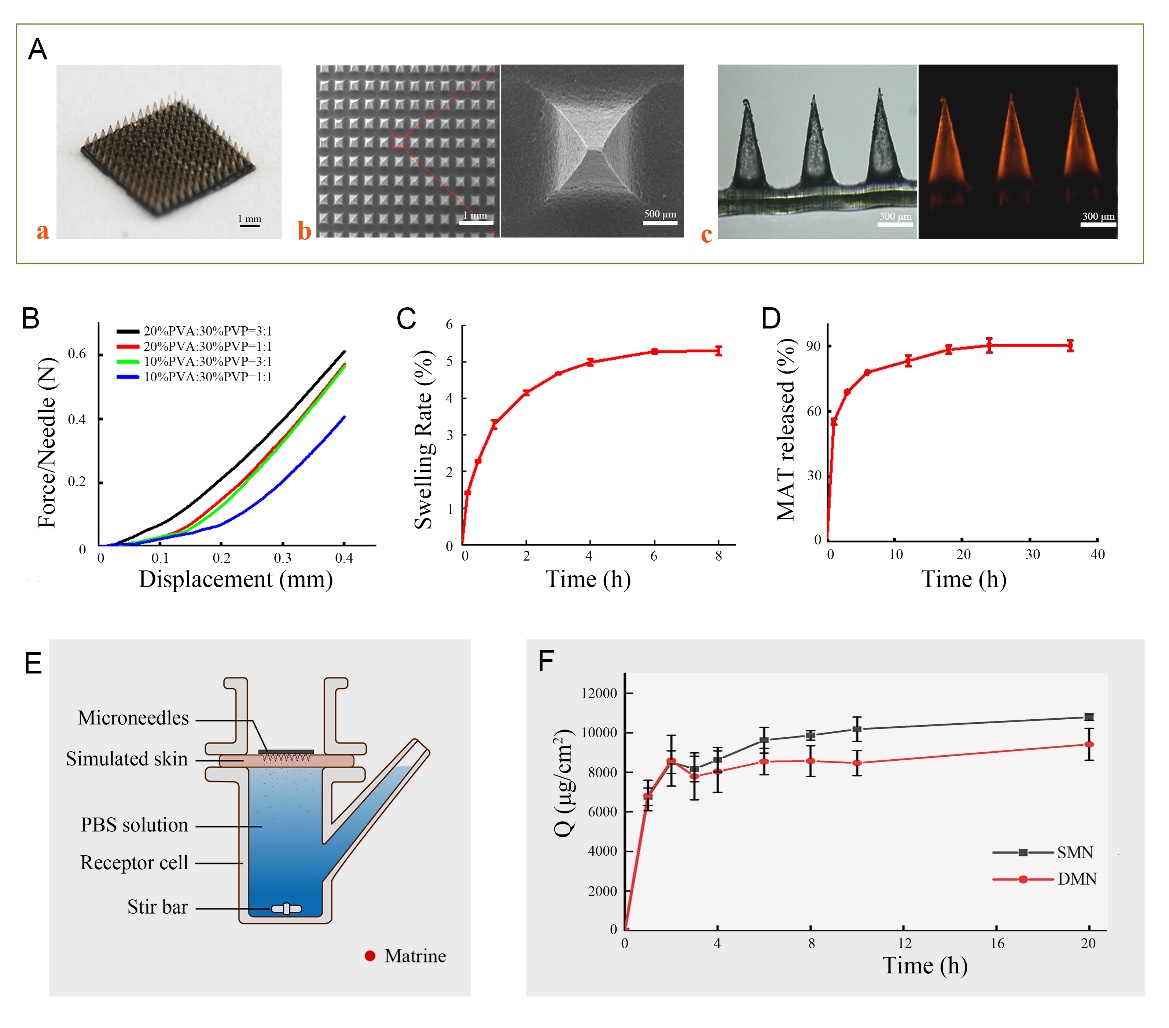
**

Fig. S1 Characterization and antibacterial activity of SDSMNs patch. (A) Representative overview image (a), SEM images (b) and microscopic images (c) of SDSMNs. (B) Mechanical strength of SDSMNs array (10 × 10 needles) characterized by axial fracture force per needle as function of displacement. (C) The swelling rate of microneedles as immersed in SBF at 37 ℃ for 8 h. (D) The in vitro release rate of MAT from MAT/SDSMNs as incubated in PBS (7.4) at 37 ℃. (E) Schematic of transdermal release using simulated skin mounted on Franz cell. (F) Cumulant drug release of PVP dissolving microneedle (DMN) and SDSMNs.


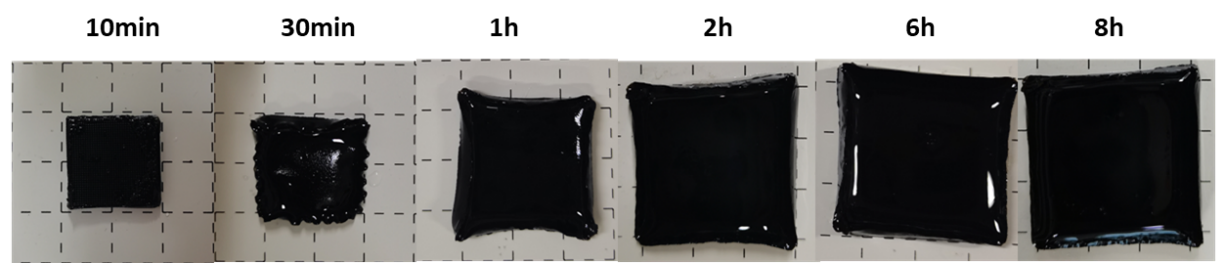


Fig. S2 Representative images of swelling performance of SDSMNs in simulated body fluid at different time


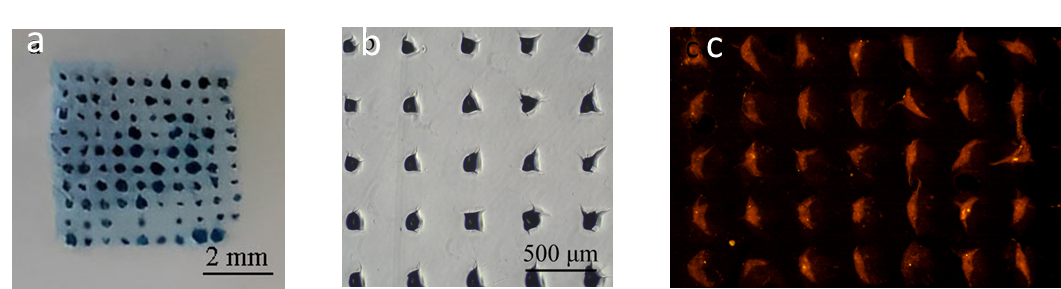


Fig. S3 Representative images of skin insertion using mice skin and simulated skin


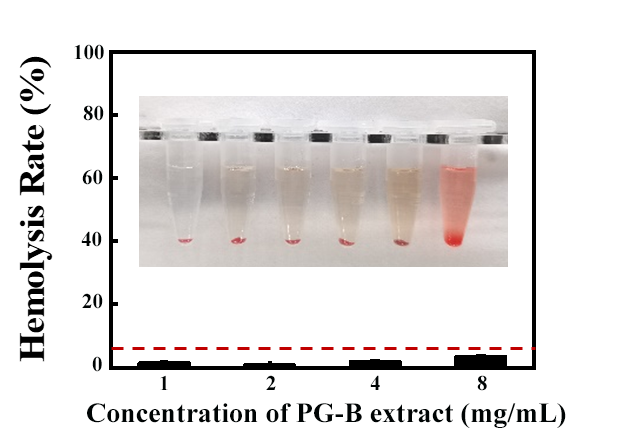


Fig. S4 Hemolysis of extract of PG-B substrate (oxidized with 4% NaIO_4_) as function of concentration.


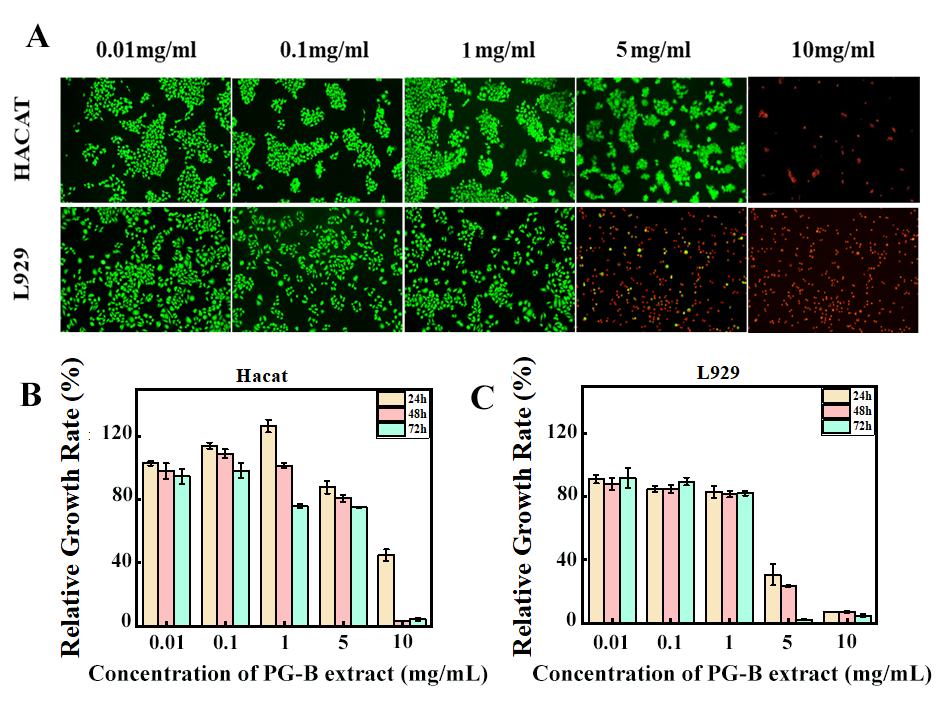


Fig. S5 Cytotoxicity of extract of PG-B substrate (oxidized with 4% NaIO_4_). (A) Representative images of Live/dead assay of L929 and HACAT after treatment with PG-B extract (0.01, 0.1, 1, 5, 10 mg/mL). The relative growth rates of HACAT (B) and L929 (C) after incubation with PG-B extract (0.01, 0.1, 1, 5, 10 mg/mL) for 24, 48, and 72 hours


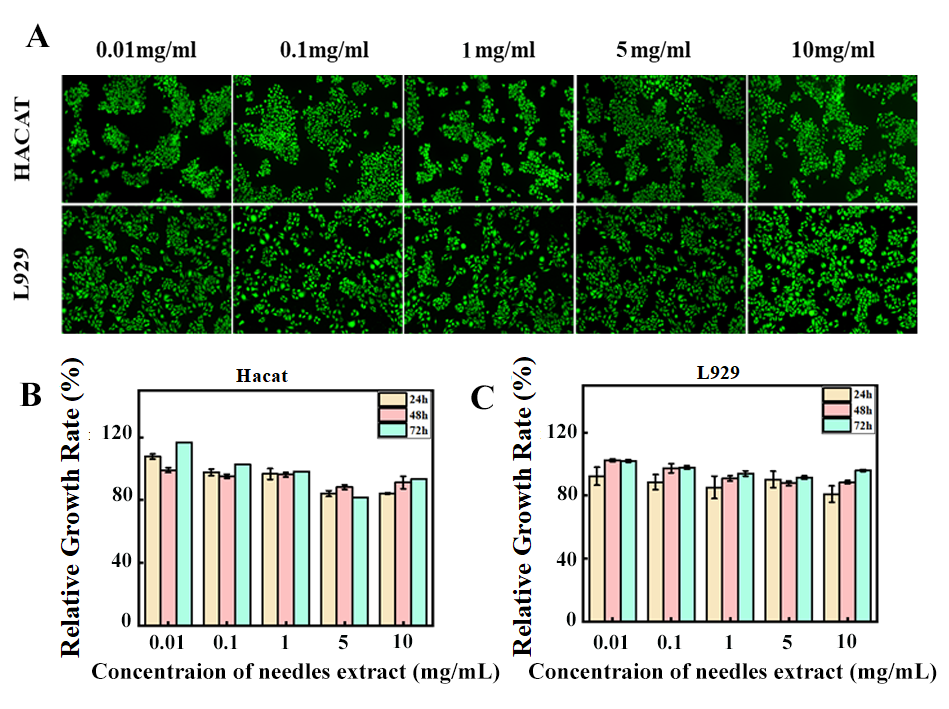


Fig. S6 Cytotoxicity of extract of needles (20% PVA: 30% PVP = 3: 1). (A) Representative images of Live/dead assay of L929 and HACAT after treatment with needles extract (0.01, 0.1, 1, 5, 10 mg/mL). The relative growth rates of HACAT (B) and L929 (C) after incubation with PG-B extract (0.01, 0.1, 1, 5, 10 mg/mL) for 24, 48, and 72 hours


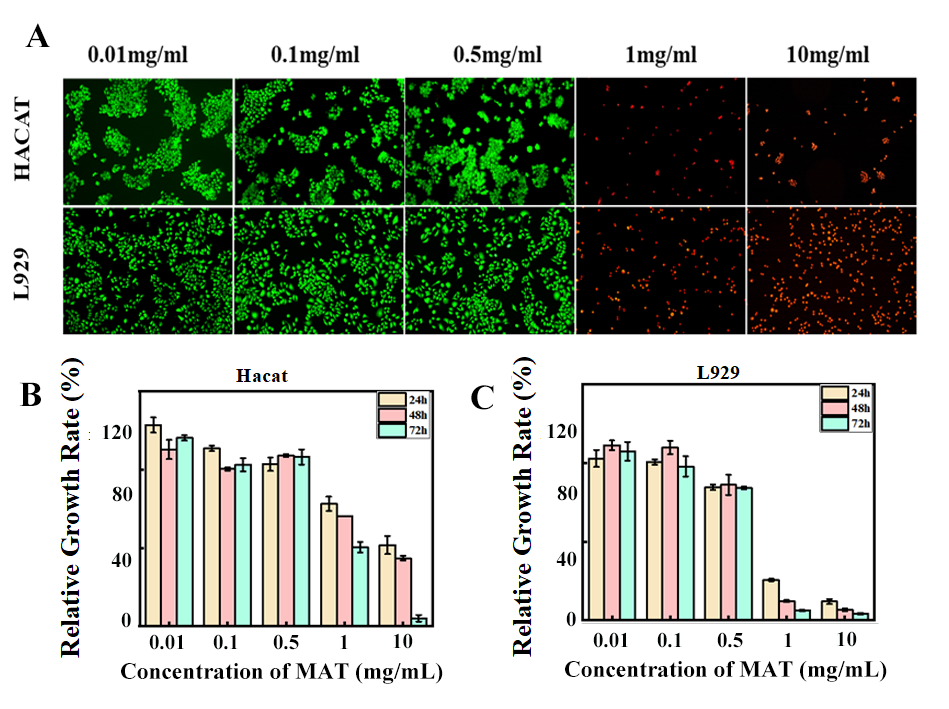


Fig. S7 Cytotoxicity of MAT. (A) Representative images of Live/dead assay of L929 and HACAT after treatment with MAT solution (0.01, 0.1, 0.5, 1, 10 mg/mL). The relative growth rates of HACAT (B) and L929 (C) after incubation with MAT solution (0.01, 0.1, 0.5, 1, 10 mg/mL) for 24, 48, and 72 hours

Table S1 Primer sequences for qRT-PCR

| **Name** | **Sequence（5'-3')** |
| --- | --- |
| M- IL-17R -Fw | CACTCACTCTACGCAACCTTAA |
| M- IL-17R -Rv | AGATGCCCGTGATGAACC |
| M- GAPDH -Fw | AACGGATTTGGTCGTCGTATTGG |
| M- GAPDH -Rv | GGGTGGAATCATATTGGAACA |

**References**

Wang M, Zhao Y, Zhang QY (2022) Human mesenchymal stem cell-derived exosomes accelerate wound healing of mice eczema. J Dermatol Treat 33:1401-1405
